# Hemodynamic Effect of the Last Finishing Coils in Packing the Aneurysm Neck

**DOI:** 10.3389/fneur.2020.598412

**Published:** 2020-11-20

**Authors:** Hailin Wan, Gang Lu, Lei Huang, Liang Ge, Yeqing Jiang, Gaohui Li, Xiaochang Leng, Jianping Xiang, Xiaolong Zhang

**Affiliations:** ^1^Huashan Hospital, Fudan University, Shanghai, China; ^2^ArteryFlow Technology Co., Ltd, Hangzhou, China

**Keywords:** hemodynamics, intracranial aneurysms, stent, coiling, recurrence

## Abstract

**Background:** Using the finishing coils to densely pack the aneurysm neck is necessary. However, the exact hemodynamic effect of finishing coils in packing the aneurysm neck is unknown.

**Objective:** To evaluate the hemodynamic characteristics of finishing coils to densely pack the aneurysm neck, using finite element method simulation.

**Methods:** A computational study was performed based on a 44-year-old female patient with an unruptured wide-necked carotid-ophthalmic artery aneurysm treated with low-profile visualized intraluminal support stent-assisted coil embolization. Four computational fluid dynamics models including pre-treatment, post-stenting, common stent-assisted coil embolization (SACE), and common SACE with finishing coils were evaluated qualitatively and quantitatively.

**Results:** Compared with the baseline of pretreatment model (100%), sac-averaged velocity in post-stenting, common SACE, and common SACE with finishing coil models decreased to 95.68%, 24.38%, and 13.20%, respectively; high flow volume (>0.1 m/s) around the aneurysm neck decreased to 92.19%, 9.59%, and 5.57%, respectively; and mean wall shear stress increased or decreased to 107%, 25.94%, and 23.89%, respectively.

**Conclusion:** Finishing coils to densely pack the aneurysm neck can generate favorable hemodynamic modifications, which may decrease the recurrence.

## Introduction

Coil embolization for intracranial aneurysms (IAs) is an effective treatment modality which is far less invasive than the long-standing convention of surgical clipping (1). However, recanalization and coil compaction after embolization is not uncommon, with recurrence rates as high as over 30% reported in the literature (2, 3). One factor that may contribute to recurrence after coiling is residual inflow in the aneurysmal sac (4). Therefore, coiling density is an important factor to predict post-coiling outcomes, and usually aneurysms are packed as densely as possible to avoid coil compaction (5–7). Stent-assisted coiling embolization (SACE) has also been found to reduce the recurrence of wide-necked aneurysm embolization (8). However, some wide-necked aneurysms after SACE can still be found recanalized especially at the aneurysm neck. Consequently, densely coiling the aneurysm neck is necessary.

The coils to pack the aneurysm sac could be divided into frame coils, filling coils, and finishing coils. However, the exact hemodynamic effect of finishing coils in packing the aneurysm neck is unknown. This study aims to investigate the hemodynamic characteristics of finishing coils to densely pack the aneurysm neck, using finite element method (FEM) simulation.

## Methods

### Computational Fluid Dynamics (CFD) Study Protocol Design

The hemodynamics of four models including pre-treatment, post-stenting, common SACE, and common SACE with finishing coils were evaluated qualitatively and quantitatively. Packing density is defined as the ratio between the inserted coils and aneurysm volume.

### Aneurysm Model

**Figure 1:**
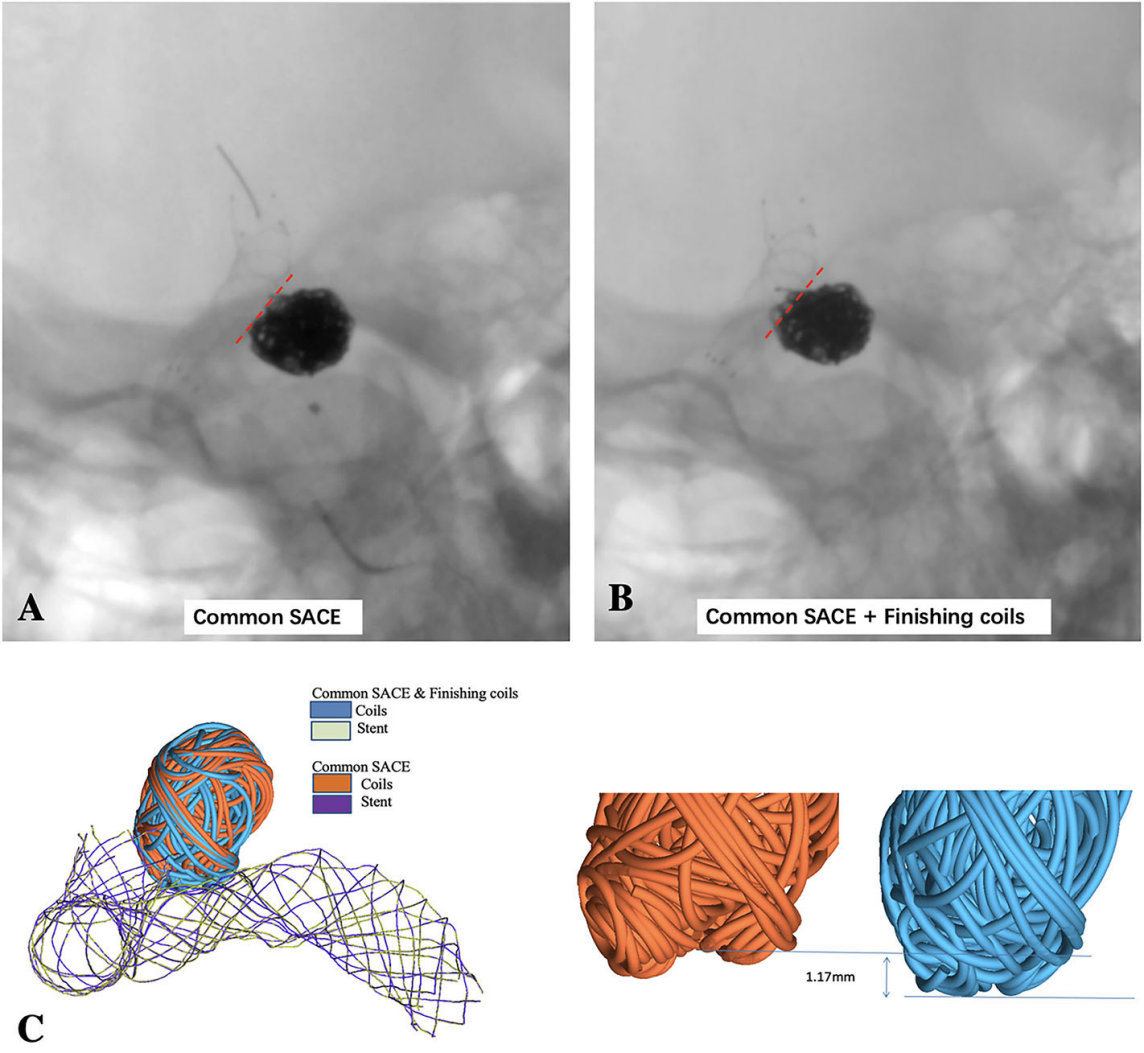
LVIS stent-assisted common coiling without deploying the last two small coils to keep the coil configuration under out the outline of the parent artery **(A)** and LVIS stent-assisted coiling with small hyper-soft coils densely coiling the aneurysmal neck **(B)** are observed in the un-subtracted angiographies; **(C)** schematic diagram of LVIS stent-assisted coiling with small coils densely packing aneurysmal neck model and common SACE model using finite element method (FEM) simulation.

A 44-year-old female patient with an incidentally found right carotid-ophthalmic artery wide-necked aneurysm (dome and neck dimension: 5.45 mm/3.37 mm) was treated with low-profile visualized intraluminal support (LVIS) stent-assisted densely coiling aneurysm neck technique and included in this CFD study (Figure 1).

The carotid-ophthalmic aneurysm was reconstructed in this study for demonstrating the hemodynamic effect of finishing coils to densely pack the aneurysm neck. 3D rotational angiography images were obtained and 3D reconstruction in surface-triangulation format and isolation of the region of interest were performed through the open source software, VMTK (www.vmtk.org). For the FEM analysis, CFD meshing, and flow simulation, a 3D segmented geometry was cleared by using Geomagic tool (Geomagic, Morrisville, NC).

### Finite-Element Method Modeling of Coiling and Stent Deployment

LVIS stent (3.5 × 15 mm; MicroVention-Terumo, Tustin, CA) was generated virtually using SolidWorks (Dassault Systemes; SolidWorks, Waltham, MA) and transformed into finite element analysis (FEA) software ABAQUS (SIMULIA, Providence, RI) to perform the stenting of aneurysm. Meanwhile, frame and helical coils were generated in MATLAB (MathWorks, Natwick, MA) and the shape of these coils was simplified by using a centerline (9). A continuous cylindrical structure in 3D shape was assumed for the coils and then the coils were swept to 3D configuration after deployed in the aneurysm sac (10, 11).

The FEA-based workflow for stent deployment modeling was performed in ABAQUS/Explicit v6.14, and the stent was modeled with nitinol super-elasticity material and the parameter values were obtained from previous studies by Reedlunn et al. (9). The simulation consists of three steps: crimping, delivery, and deployment. The crimping of stent was performed and used for the initial condition for the delivery process through the predefined field tool in ABAQUS. A delivery path was generated with central points of the cross-sections of the blood vessel and the crimped stent within microcatheter was delivered through the path to the orifice of the aneurysm according to the process of delivery of a stent during clinical treatment. The crimped stent was assembled in a microcatheter in the global coordinate system and delivered to the aneurysm orifice of the pre-treated model through a displacement load according to the central points of the arterial wall along the delivery path. With the predefined stress–strain field, the stent was released in the next step. A “general contact” algorithm in ABAQUS was used for the complex interactions during the stent delivery and deployment procedures, with a friction coefficient value of 0.15.

After the stent deployment, SACE with small hyper-soft coils densely packing the aneurysm neck model was created after 11 coils including one 6 mm × 20 cm coil, one 5 mm × 20 cm coil, one 3 mm × 8 cm coil, two 3 mm × 6 cm coils, one 1.5 mm × 4 cm coil, four 1.5 mm × 3 cm coils, and one 1 mm × 3 cm coil (MicroPlex-10; MicroVention, Aliso Viejo, CA). All the coils were swept to 3D solid model using ABAQUS/CAE with the real diameter of the coils and were then successively deployed into the aneurysm. Common SACE model was built without deploying the last two hyper-soft coils to keep the configuration of the previous coils under out of the outline of adjacent parent artery according to the actual operation (Figure 1B). To the end, the surface-based aneurysm and vessel model with the 3D representation of coils and the stent was subsequently used for the CFD analysis. The detailed process and methods in simulation of patient-specific endovascular stenting and coiling for intracranial aneurysm using FEM can be found in our previous study (12).

### CFD Simulation

The aneurysm model was meshed with polyhedral grids and four-layer wall prism elements (for accurate boundary layer resolution) consisting of ~2 million elements for pre-treatment model and up to 20 million cells for SACE model using ICEM-CFD meshing tool (Ansys, Canonsburg, PA). Incompressible Navier–Stokes equations under steady flow conditions was used to obtain the numerical solution by using the finite volume CFD solver, CFX V19 (Ansys, Canonsburg, PA). The mean flow rate for ICA inlet was 4.6 ml/s and used as inlet boundary conditions. Traction-free boundary conditions were applied at the outlet and the mass flow rate through each outlet vessel was set to be proportional to the cube of its diameter based on the principle of optimal work (13). With a density of 1,056 kg/m^3^ and a dynamic viscosity of 0.0035 N·s/m^2^, the blood was modeled as a Newtonian fluid material. The vessel walls were modeled as a rigid wall with no-slip boundary conditions.

For qualitative analysis, the aneurysmal flow streamlines, iso-velocity surface (high flow region around the neck plane), and wall shear stress (WSS) were analyzed. Taking the pre-treatment model as the baseline (100%), sac-averaged velocity, high velocity regions, and sac-averaged WSS were analyzed quantitatively. High velocity regions were defined where flow velocity magnitudes are larger than 0.1 m/s. WSS indicated the friction force between blood and inner surface of arterial wall, which was found to have an essential influence on aneurysm initiation, growth, and rupture (14, 15).

## Results

### Qualitative Analysis of CFD

**Figure 2:**
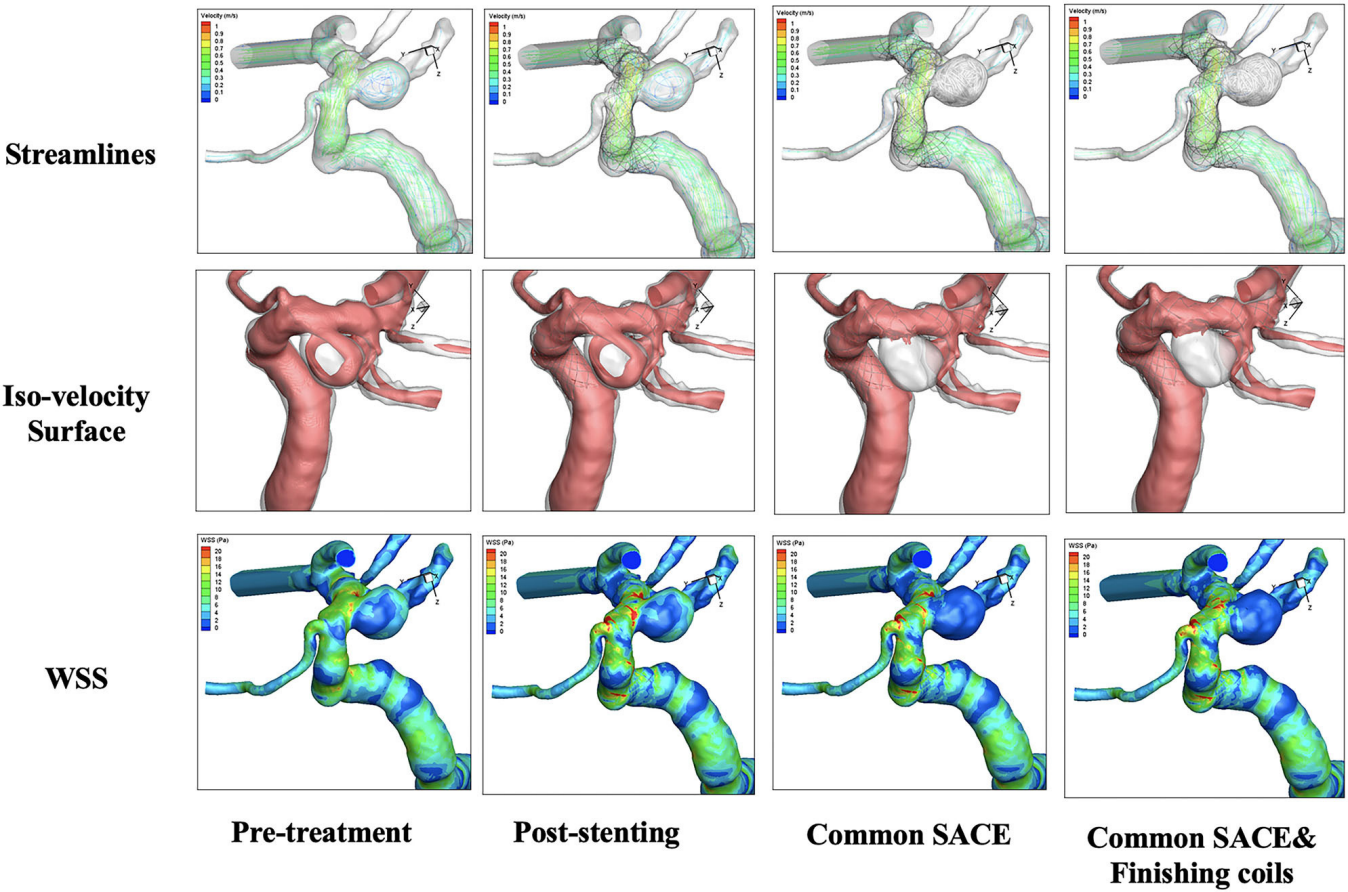
Qualitative comparison was performed in the four models including pre-treatment, post-stenting, common SACE, and common SACE with finishing coils.

CFD simulations for four models were performed with color-coded streamlines, iso-velocity surface, and WSS, respectively (Figure 2). For flow streamlines, high flow volume via iso-velocity surface, and WSS in the aneurysmal sac, compared with the pre-treatment model, the other models decreased dramatically except the stenting model. In the four models, the reduction in high flow regions around the common SACE with finishing coils model was mildly stronger than for common SACE, whereas WSS in aneurysmal sac was comparable with that of common SACE model.

### Quantitative Analysis of CFD

**Figure 3:**
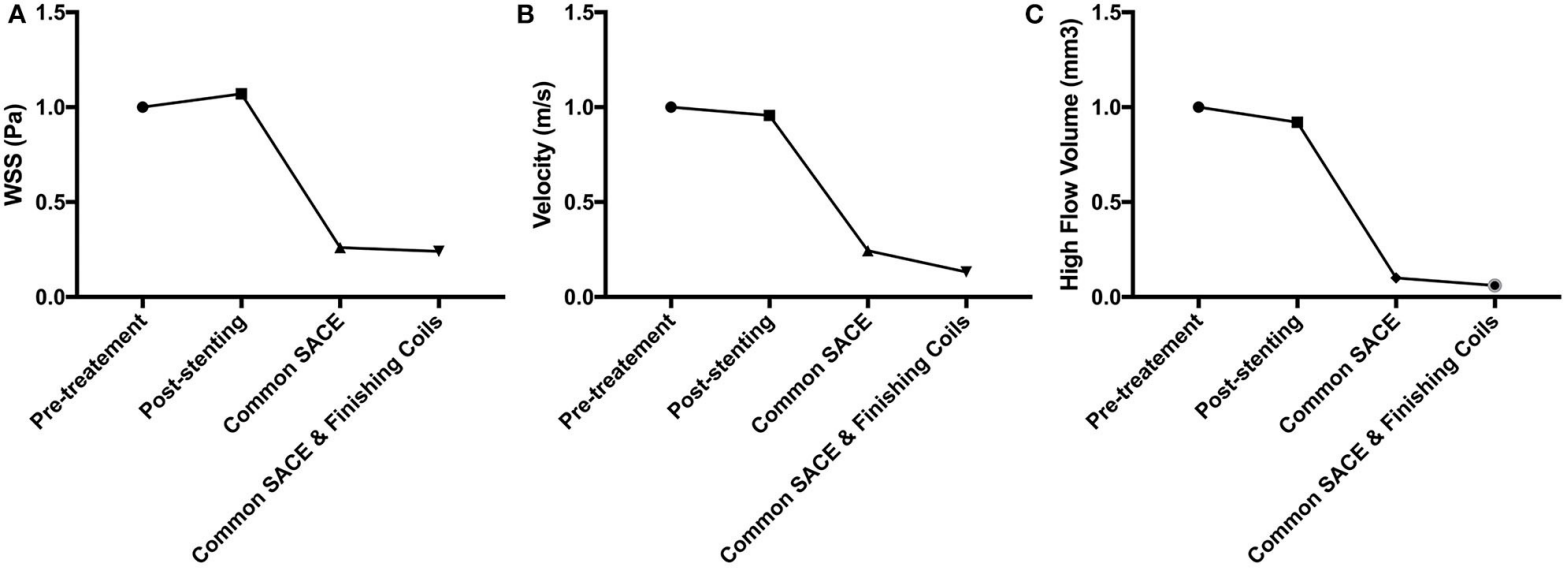
CFD simulation results for WSS magnitude **(A)**, average velocity **(B)**, and high flow volume (>0.1 m/s) **(C)** in the aneurysm for the four models were compared quantitatively.

The aneurysmal volume was 123.61 mm^3^ and the packing density is 33.49% in the common coiling models. Compared with the baseline of pretreatment model (100%), sac-averaged velocity in stenting, common SACE, and common SACE with finishing coil models decreased to 95.68%, 24.38%, and 13.20%, respectively; high flow volume (>0.1 m/s) around the aneurysm neck decreased to 92.19%, 9.59%, and 5.57%, respectively; and mean WSS increased or decreased to 107%, 25.94%, and 23.89%, respectively (Figure 3).

## Discussion

The present study simulated the procedure of the LVIS stent-assisted coil embolization in a carotid-ophthalmic aneurysm and evaluated its CFD characteristics qualitatively and quantitatively. Two main findings were obtained from our numerical modeling and data analyses. First, this study is the first to simulate LVIS SACE with small hyper-soft coils densely packing the sidewall aneurysm neck using finite element methods. Second, common SACE with finishing coil model substantially reduced the flow for both sac-averaged velocity and high flow volume compared with common SACE. To our knowledge, this study is the first to evaluate the hemodynamic characteristics of last finishing coils based on CFD simulation and provide a proof of concept of hemodynamic modifications resulting from small hyper-soft coils densely coiling the aneurysm neck.

It has been hypothesized that the decreased blood flow in the aneurysmal sac induced by coiling may initiate thrombosis, subsequent clot formation, and fibrosis (16). Residual blood flow in the aneurysmal sac prevents thrombus organization and endothelial cell proliferation across the neck, which may lead to eventual aneurysmal recanalization (17, 18). In the present study, the finishing coils could induce substantial reduction, especially in the region of aneurysmal neck, which might facilitate thrombosis and decrease the recurrence rate. In clinical practice, Wan et al. found that embolization of aneurysm neck was an effective and safe modality for the ruptured aneurysms with bleb formation (19).

Interestingly, in the post-stenting model, the mean velocity of aneurysmal sac and high flow volume around the neck just decreased which is consistent with previous studies (20, 21). However, the WSS increased to 107% of pre-treatment model, which may be due to the high jet flow through the stent mesh. In another aspect, it might indicate that the LVIS stent not only can divert flow but also can generate unfavorable hemodynamic effect.

In this study, common SACE with finishing coil model performed better in aneurysmal hemodynamic modification compared with common SACE model. These findings were obtained by qualitative observations and quantitative data analyses of CFD simulation. Velocity and high flow volume reductions within the aneurysm were observed in the all models, consistent with previous CFD-based studies (22–25). Of particular note, in this study high flow volume (>0.1 m/s) around the aneurysmal neck plane was significantly reduced in common SACE with finishing coil model. Furthermore, the high flow volume in the common SACE with finishing coils model was almost only half that of common SACE model. Babiker et al. (4) revealed that increased coiling density was accompanied with reduced cross-neck flow rate. Furthermore, Morales et al. (22) found that coil configuration initially played an important role in intra-aneurysmal hemodynamics until a high coiling density (nearly 30%) was achieved. Sluzewski et al. (6) reported that in aneurysms with coiling density between 20 and 23.9%, compaction did not occur if the aneurysm volume was <200 mm^3^. Because the packing density (33.49%) in this study is high, consequently it was unnecessary to evaluate coil configuration in this study, while the aneurysmal volume was ~124 mm^3^.

Several limitations in this study should be noted. First, this one-sample proof-of-concept study just represents this innovative technique. Further prospective studies with greater sample sizes and longer follow-up are needed to verify our study results. Second, this technique is solely appropriate for wide-necked sidewall aneurysms with median size. For the large or giant sidewall aneurysms, flow diverter or parent artery occlusion may be optimal alternative modalities. Third, we adopted several commonly used assumptions to make CFD tractable. Due to a lack of patient-specific information, we assumed a constant, location-based inlet flow rate. Inlet velocities were scaled according to the inlet diameter. This study utilized the pre-treatment model as a baseline and evaluated the relative, not absolute, hemodynamic change.

## Conclusion

This study demonstrated the last finishing coils to densely pack the aneurysm neck could generate more favorable hemodynamic modifications compared with common SACE, which can accelerate the thrombus formation in the aneurysmal neck and likely decreases the recurrence risk.

## Data Availability Statement

The raw data supporting the conclusions of this article will be made available by the authors, without undue reservation.

## Ethics Statement

The studies involving human participants were reviewed and approved by Huashan Hospital, Fudan University. The patients/participants provided their written informed consent to participate in this study.

## Author Contributions

XZ and JX had the idea for the article. HW, GLi, and XL performed the computational fluid study. HW, LH, GLu, LG, and YJ performed the literature search. HW wrote the article. XZ and JX are the guarantors. All authors contributed to the article and approved the submitted version.

## Conflict of Interest

GLi, XL, and JX were employed by the company ArteryFlow Technology Co., Ltd. The remaining authors declare that the research was conducted in the absence of any commercial or financial relationships that could be construed as a potential conflict of interest.
